# Supplementary material for: Hybrid Models and Biological Model Reduction with PyDSTool
Source: PLoS Comput Biol. 2012 Aug 9;8(8):e1002628. doi: 10.1371/journal.pcbi.1002628 (PMC3415397; doi:10.1371/journal.pcbi.1002628)
Supplement: Text S4 — Complete source code for the PyDSTool package (version 0.88.120504). Includes API documentation and help files linking to web pages. This file is identical to the current public release on Sourceforge.net. (ZIP) [file pcbi.1002628.s004.zip › PyDSTool/html/toc-PyDSTool.Points-module.html]

xml version="1.0" encoding="ascii"?


Points


# Module Points

---

## Classes

Point  
 PointInfo  
 Pointset  
 VarCaller  

## Functions

\_pretty\_print\_label

arrayToPointset  
 comparePointCoords  
 exportPointset  
 export\_pointset\_to\_CSV  
 importPointset  
 isparameterized  
 makeNonParameterized  
 mergePointsets  
 padPointset  
 pointsToPointset  

## Variables

\_1DimplicitSolveMethods

\_all\_complex

\_all\_float

\_all\_int

\_all\_numpy\_complex

\_all\_numpy\_float

\_all\_numpy\_int

\_complex\_types

\_float\_types

\_implicitSolveMethods

\_int\_types

\_num\_equivtype

\_num\_maxmin

\_num\_name2equivtypes

\_num\_name2type

\_num\_type2name

\_num\_types

\_pytypefromtype

\_real\_types

\_seq\_types

point\_keys

---

[hide private]
